# Coprecipitation with Ferrihydrite Inhibits Mineralization of Glucuronic Acid in an Anoxic Soil

**DOI:** 10.1021/acs.est.3c01336

**Published:** 2023-06-09

**Authors:** Laurel K. ThomasArrigo, Sophie Vontobel, Luiza Notini, Tabea Nydegger

**Affiliations:** Soil Chemistry Group, Institute of Biogeochemistry and Pollutant Dynamics, Department of Environmental Systems Science, ETH Zurich, Universitätstrasse 16, Zurich, CHN CH-8092, Switzerland

**Keywords:** organic carbon, anoxic soils, mineralization, iron minerals

## Abstract

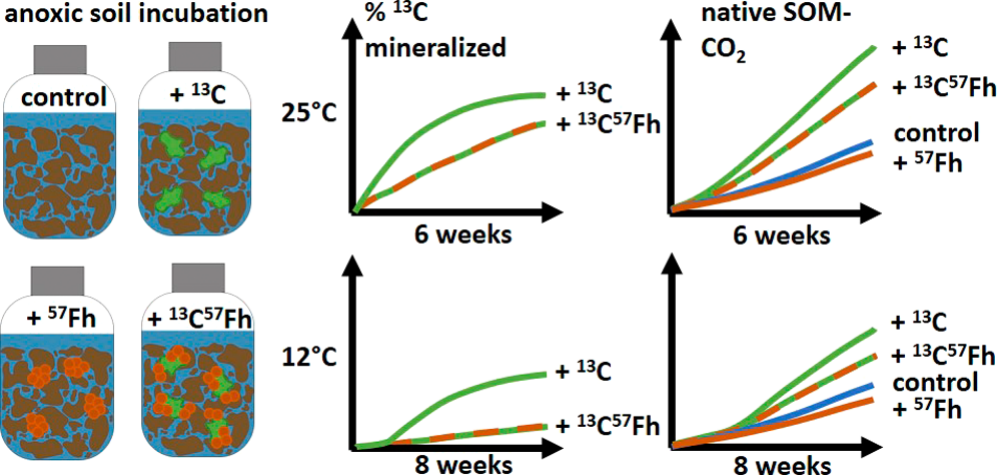

It is known that the association of soil organic matter (SOM) with iron minerals limits carbon mobilization and degradation in aerobic soils and sediments. However, the efficacy of iron mineral protection mechanisms under reducing soil conditions, where Fe(III)-bearing minerals may be used as terminal electron acceptors, is poorly understood. Here, we quantified the extent to which iron mineral protection inhibits mineralization of organic carbon in reduced soils by adding dissolved ^13^C-glucuronic acid, a ^57^Fe-ferrihydrite-^13^C-glucuronic acid coprecipitate, or pure ^57^Fe-ferrihydrite to anoxic soil slurries. In tracking the re-partitioning and transformation of ^13^C-glucuronic acid and native SOM, we find that coprecipitation suppresses mineralization of ^13^C-glucuronic acid by 56% after 2 weeks (at 25 °C) and decreases to 27% after 6 weeks, owing to ongoing reductive dissolution of the coprecipitated ^57^Fe-ferrihydrite. Addition of both dissolved and coprecipitated ^13^C-glucuronic acid resulted in increased native SOM mineralization, but the reduced bioavailability of the coprecipitated versus dissolved ^13^C-glucuronic acid decreased the priming effect by 35%. In contrast, the addition of pure ^57^Fe-ferrihydrite resulted in negligible changes in native SOM mineralization. Our results show that iron mineral protection mechanisms are relevant for understanding the mobilization and degradation of SOM under reducing soil conditions.

## Abstract 2

The effectiveness of mineral protection on mineralization of organic carbon in anoxic soils is not clear. This study shows that coprecipitation of organic carbon with iron minerals significantly reduces mineralization of the organic substrates.

## Introduction

Soils are a major terrestrial carbon pool, storing an estimated 3500–4800 Pg of soil organic carbon (OC) globally.^1^ Thus, understanding carbon cycling in soils is critical to accurately predicting global carbon cycles. In soils and sediments, the storage and mobility of soil organic matter (SOM) are influenced by dynamic adsorption and complexation interactions with minerals.^2,3^ Mineral-associated organic matter (MAOM) forms through sorption of dissolved organic carbon (DOC) to existing minerals in the soil matrix or in soil solution, or through coprecipitation of dissolved OC with newly formed minerals.^4^ The latter occurs primarily at redox interfaces, where oxidation of ferrous iron (Fe(II)) to ferric iron (Fe(III)) and its rapid hydrolysis lead to the precipitation of short-range-ordered (SRO) iron minerals in the presence of dissolved OC, forming Fe(III)–OC coprecipitates.^5−7^ MAOM is thought to be protected from biodegradation via stable chemical bonds formed in organo-metal complexes and by sorption to and occlusion within mineral aggregate structures,^4,6,8−12^ most notably involving SRO iron minerals (e.g., ferrihydrite and nanogoethite) and aluminosilicates (e.g., allophane and imogolite) whose high specific surface areas result in an abundance of surface sorption sites. Hence, the importance of soil mineral content as an indicator for OC storage in soils has gained increasing interest in the last decades,^10,13−15^ and organic-associated iron and aluminum are considered critically important to the long-term stabilization of SOM.^4,6,8,9,11,15^

Iron-bound carbon represents a high amount of total carbon in soils and sediments,^6,15,16^ storing up to 40% of total soil carbon under oxic conditions.^15,16^ In agreement, the effectiveness of iron mineral protection of adsorbed OC under oxic soil conditions has been consistently demonstrated in aerobic soil incubations,^12,17,18^ where mineralization of MAOM has been shown to be reduced by >99.5%^12^ and substrate sorption in soils with high clay content (<0.002 mm size fraction) has been shown to limit substrate mineralization.^19,20^ However, in the absence of O_2_, Fe(III) acts as a terminal electron acceptor for microorganisms during anaerobic respiration of organic matter.^21,22^ Electron transfer reactions induce the reductive dissolution, recrystallization, or transformation of iron minerals,^23,24^ with direct implications for the solubilization and mineralization of MAOM. The reductive dissolution of Fe(III) in mineral aggregates or in Fe(III)–OC coprecipitates releases adsorbed or occluded OC to soil solution.^25−30^ Mobilized OC, found as DOC^31,32^ or in organic-Fe/Al colloids,^33,34^ may further form stable complexes with dissolved Fe,^35^ be transported to deeper soil horizons,^36^ or may be subsequently mineralized.^37^ Moreover, microbial use of Fe(III) as an electron acceptor can directly result in CO_2_ production through the metabolic coupling of OC oxidation to Fe(III) reduction.^22,38^ Thus, under reducing soil conditions, mineralization of SOM can even be stimulated by the addition of Fe(III) minerals acting as electron acceptors.^39,40^ In anoxic soils, microbial Fe reduction may account for up to 44% of anaerobic OC mineralization.^41^

Yet, as demonstrated in the millennial-scale age of Fe-bound OC found buried in marine sediments^42^ and through abundant examples of very long turnover times for mineral-bound SOM in subsurface soil horizons,^10,43^ evidence overwhelmingly agrees that significant stores of Fe-bound OC exist in soil and sediment environments which experience continual or recurring reducing conditions. This disconnect, which suggests that mechanisms of iron mineral protection are similarly prevalent in reducing soil environments, can be explained through a lack of mechanistic understanding of mineral protection of OC in reducing soil environments; a critical knowledge gap which currently limits our ability to predict the stabilization or mobilization of MAOM in reducing or redox-active soil environments.

In this study, we quantified the extent to which iron mineral protection inhibits the mineralization of MAOM, modeled here as a Fe(III)–OC coprecipitate, in a reducing soil environment. To this end, soil slurries were amended with either dissolved ^13^C-glucuronic acid (^13^GluC) or a ^57^Fe-ferrihydrite-^13^C-glucuronic acid coprecipitate (^57^Fh^13^GluC) and incubated under anoxic conditions for 6 weeks at 25 °C or 8 weeks at 12 °C. The fate of the added ^13^C-glucuronic acid was followed through its re-partitioning to the aqueous and solid phases and through its transformation to ^13^CO_2_. Furthermore, to understand the extent to which mineral association impacts soil priming effects resulting from the ^13^C-glucuronic acid additions, total CO_2_ derived from mineralization of native SOM in the ^13^GluC and ^57^Fh^13^GluC treatments were compared to soil slurries amended with solely ^57^Fe-ferrihydrite (^57^Fh) and to a control treatment which received neither additional Fe nor C. In the 25 °C treatments, trends in aqueous geochemical conditions, including Eh, pH, aqueous Fe, and DOC, were recorded, and microbial reduction of Fe(III) was tracked through changes in acid-extractable solid-associated Fe(II) and by following the iron isotope composition of the aqueous phase. Collectively, the results of this study demonstrate that sorption and coprecipitation, mechanisms of iron mineral protection of organic matter widely accepted to occur in oxic soil environments, are similarly prevalent under reducing soil conditions and limit mineralization of MAOM over timescales of weeks, a finding which holds implications for understanding present and future trends in carbon cycling in reduced and redox-active terrestrial environments.

## Materials and Methods

### Study Site

For this study, soil from a Gleyic Andosol (GA; Icelandic soil classification system), a typical soil type found across north and western Iceland,^44^ was included. Specifically, a subsoil horizon (60–72 cm depth) from the Hestur_GA soil profile,^7,34^ located in the Borgarfjördur catchment in western Iceland (Figure S1), was chosen. The characterization of horizons from this soil profile was included in a previous publication.^34^ For this study, fresh soil samples were collected in July 2020. Details about the study site, soil sampling, and characterization are presented in the Supporting Information. Briefly, soil pH was 4.56, and total Fe and C contents were 73.1 mg g^–1^ and 21.6 wt %, respectively. X-ray diffraction patterns indicated the presence of plagioclase feldspars, pyroxenes, and small contributions from quartz and contained a significant amorphous fraction (Figure S2 and Table S2).

### Mineral Synthesis and Characterization

All solutions used in this experiment were prepared from ultra-pure water (UPW, Milli-Q, Millipore, 18.2 MΩ·cm). The synthesis of isotope-labeled ferrihydrite (^57^Fh) and the ferrihydrite-glucuronic acid coprecipitate (^57^Fh^13^GluC) followed previously published methods^45−47^ with modifications included to enable synthesis of ^57^Fe-labeled minerals from Fe(0) metal powder. Glucuronic acid is a low molecular weight organic acid with a single carboxyl group (Figure S3). Being a derivative of glucose, a high energy substrate that can be rapidly utilized by soil microorganisms,^48^ mineralization of glucuronic acid is expected to be similarly rapid. Details on mineral synthesis and characterization of the resulting solid phases, including total element content, the fraction of easily desorbed C in the coprecipitate, and confirmation of the mineral phases present using powder XRD, can be found in the Supporting Information. Briefly, the C/Fe molar ratio of the ferrihydrite-glucuronic acid coprecipitate ^57^Fh^13^GluC was 0.42 and ∼10 mg g^–1^ C was easily desorbed, accounting for ∼22% of the total C in the coprecipitate. For both ^57^Fh and ^57^Fh^13^GluC, XRD patterns confirmed the presence of 2-line ferrihydrite, visible as broad maxima around 2.54 and 1.49 Å (Figure S4).

### Soil Slurry Incubation

Prior to starting the experiment, the field-moist soils were sieved to <2 mm with a nylon sieve, and visible plant or root material was removed with tweezers. The prepared soils were then packaged into plastic bags and kept at 25 or 12 °C in the dark for two weeks to allow soil microorganisms to recover from 4 °C storage. Soil incubations were conducted as soil slurries at a soil/water ratio of 1:10 in Al-wrapped septum bottles. Four treatments were considered: soil amended with ^57^Fh, soil amended with ^57^Fh^13^GluC, soil amended with ^13^GluC, and a control with no amendments. For the 25 °C incubation, two complete sets of triplicate treatments were prepared to allow for (1) destructive sampling of the soil slurry and (2) repeated sampling of headspace gasses only (Table S3). To this end, the soil (6.82 or 2.92 g, equivalent to 3.5 or 1.5 g of dry soil, respectively) was added to 117 mL or 58 mL septum bottles (respectively), which were then moved into an anoxic glovebox (MBRAUN, N_2_ atmosphere, <1 ppm (v/v) O_2_) and covered in Parafilm (to allow gas exchange but prevent evapotranspiration). After 24 h, anoxic UPW (30.2 or 12.1 mL, respectively) was added, followed immediately by the amendment spikes. To this end, the synthesized coprecipitates ^57^Fh and ^57^Fh^13^GluC or ^13^GluC were resuspended in 1.5 mL of anoxic UPW directly prior to spiking to the septum bottles. The control treatment received 1.5 mL of UPW. Immediately following the amendment spikes, the bottles were crimp-sealed with rubber stoppers and removed from the glovebox. To ensure sufficient headspace gas for sampling and measurements (detailed below), the 58 mL septum bottles were incubated at an initial overpressure of ∼700 mbar. To this end, 30 mL of humidified N_2_ gas was injected into the headspace through a needle connected to a syringe with a 3-way stopcock valve. All treatment bottles were then placed on an orbital shaker (150 rpm) in a temperature-controlled room at 25 °C. Because the soil used in this study originates in Iceland, where average high temperatures recorded during the summers are near 11 °C,^44^ we conducted an additional soil slurry incubation including the same soil and treatments at 12 °C for 8 weeks to assess whether the trends in SOM mineralization and mineral protection of organic substrates seen in our 25 °C experiment are transferable to high latitude soils. For the 12 °C incubation, additional sample bottles containing 3 g of dry soil equivalent were prepared as described above in duplicate in 58 ml septum bottles. Amendment spike ratios in the 12 °C incubations are presented in detail in Table S4.

### Soil Slurry Sampling

To prevent significant accumulation of CO_2_ in the headspace of the 117 mL septum bottles, the headspace was purged with humidified N_2_ gas at a flow rate of 750 mL min^–1^ for 10 min every 2–4 days during the entire experiment. During purging, the bottles were placed on an orbital shaker (150 rpm) at room temperature. Aqueous geochemical parameters were regularly measured in the 25 °C incubation experiment. To this end, after 72 h and 1, 2, 4, 5, and 6 weeks following the purging of the headspace, the 117 mL septum bottles were moved into the glovebox and opened for anoxic sampling. First, pH and Eh were measured directly in the soil slurry. The bottles were then manually agitated to ensure resuspension of all soil particles, and ∼5 mL of the soil slurry was poured into 15 mL Falcon tubes which were then capped, wrapped in Parafilm, and removed from the glovebox for centrifugation (3000 *g* for 15 min). The centrifuged tubes were returned to the glovebox, the supernatant pipetted off, , filtered (<0.45 μm, nylon), and acidified for further aqueous analyses (described below). To ensure the removal of all aqueous Fe(II), the residual solid phase was then resuspended by adding 5 mL of anoxic UPW to the Falcon tube and manually shaking it. The Falcon tubes were then again capped and centrifuged (as described above) and returned to the glovebox, where the supernatant pipetted off, and the residual solid phase allowed to dry in the glovebox atmosphere (<24 h). After sampling, the reaction bottles were re-crimp-sealed, removed from the glovebox, and returned to the orbital shaker (150 rpm) at 25 °C.

### Aqueous- and Solid-Phase Analyses

Filtered aqueous samples were measured for total element contents with inductively coupled plasma–optical emission spectrometry (ICP–OES, Agilent 5100) and Fe isotope composition by inductively coupled plasma mass spectrometry (ICP–MS, Agilent 8800 Triple Quad), as previously described.^45,46^ Iron isotope composition results are reported as *f*^*n*^Fe, whereby the counts per second (cps) of the isotope of interest *n* (=56 or 57) is divided by the sum cps of the Fe isotopes ^56^Fe and ^57^Fe. In previous anoxic incubations of Icelandic wetland soils, aqueous Fe was shown to comprise primarily Fe(II);^34^ therefore, aqueous Fe (Fe_aq_) in this study is assumed to similarly comprise Fe(II). Dissolved organic carbon (DOC) in the filtrates was measured with a Dimatoc 2000 TOC analyzer (Dimatec). For treatments containing ^13^C-glucuronic acid (^57^Fh^13^GluC and ^13^GluC), aqueous and solid samples collected at 2, 4, and 6 weeks were additionally measured for their C isotope composition on an OI Aurora 1030W DOC-DIC system linked to a ThermoFisher Scientific Delta V plus isotope ratio mass spectrometer (IRMS)^49^ and a ThermoFisher Flash-EA 1112 coupled with a Conflo IV interface to a ThermoFisher Delta V IRMS, respectively. Further details on these analyses are found in the Supporting Information. Isotope ratios are reported in the conventional δ-notation with respect to the Vienna Pee Dee Belemnite (V-PDB) standard
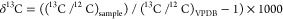
1

Acid-extractable solid-associated Fe(II) was solubilized by resuspending the solid-phase sample in 0.5 M HCl and shaking it for 2 h on a horizontal shaker at 150 rpm in the glovebox. The extracts were then centrifuged (18620 rcf for 10 min), and the supernatant was carefully pipetted off. The amount of solid-associated Fe(II) was then estimated by measuring Fe(II) in the extracts with the 1,10-phenanthroline method.^50,51^

### Headspace Gas Sampling and Measurements

At selected timepoints, headspace gasses from the 25 and 12 °C incubations were sampled for measurements of CO_2_ and CH_4_ and their δ^13^C values with gas chromatography (8610c, SRI Instruments) and cavity ring-down spectrometry (CRDS, Picarro G2201-I) using a Small Sample Introduction Module (SSIM, Picarro A0314). Details on these measurements and calculations of dissolved CO_2_ are reported in the Supporting Information.

The percent contribution of the ^13^C-glucuronic acid-derived CO_2_ (^13^GluC-CO_2_) to total CO_2_ respired (C_GluC_) was estimated using a two-source mixing model^52^
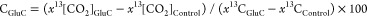
2where *x*^13^[CO_2_]_GluC_ is the atom fraction of ^13^C of CO_2_ respired in the ^57^Fh, ^57^Fh^13^GluC, and ^13^GluC treatments and *x*^13^[CO_2_]_Control_ is the atom fraction of ^13^C of CO_2_ respired in the control treatment. *x*^13^C_GluC_ is the initial atom fraction of ^13^C in the ^13^C-glucuronic acid, and *x*^13^*C*_Control_ is the initial atom fraction of ^13^C in the soil. The fraction of CO_2_ derived from native SOM (SOM-CO_2_) contributing to total CO_2_ respired (*C*_SOM_) was calculated by difference
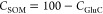
3

Statistical analyses of the effects of ^57^Fe-ferrihydrite and/or ^13^C-glucuronic acid additions on the production of SOM-CO_2_ and differences in the iron isotope composition of Fe_aq_ in the ^57^Fh and ^57^Fh^13^GluC treatments were assessed using pairwise t-tests, and differences were considered significant at *p* < 0.05. A two-way ANOVA (Tukey’s HSD) was used to assess the effects of coprecipitation on ^13^C-glucuronic acid mineralization in the ^57^Fh^13^GluC and ^13^GluC treatments over time. Statistical analyses were performed in the R software.

## Results and Discussion

### Coprecipitation Limits Glucuronic Acid-Induced Stimulation of Microbial Iron Reduction

**Figure 1:**
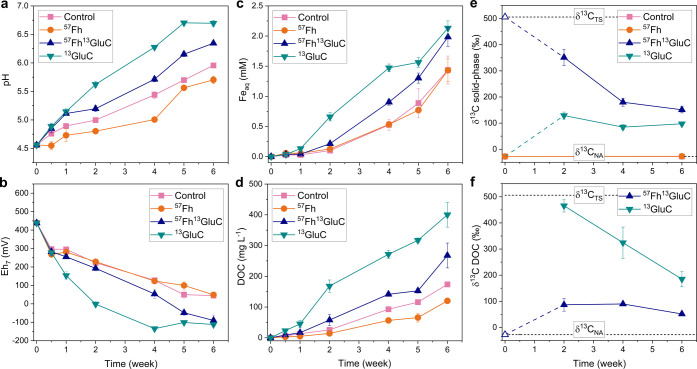
Aqueous geochemical data for the 25 °C incubations. Trends in pH (a), redox potential (Eh_7_; Eh calculated relative to pH 7) (b), aqueous Fe (Fe_aq_; panel c), and dissolved organic carbon (DOC; panel d) concentrations. Carbon isotope composition, shown as δ^13^C, of the solid-phase samples (e) and DOC (f). Horizontal dashed lines in panels (e,f) indicate the calculated isotope composition of the total system (TS) following the addition of the isotope-labeled (co)precipitates and the isotope composition of the ^13^C isotope free system (NA = natural abundance), as determined by δ^13^C measurements of the solid-phases from the control and ^57^Fh treatments (panel e). Because labile substrates added to soils under oxic conditions are rapidly mineralized (<30 s),^48^ the initial (time = 0 week, open symbols) isotope compositions of the ^13^C-labeled glucuronic acid amended treatments are calculated based on the experimental set-up (Table S3), and dashed lines are included to aid in visual interpretation. Assuming DOC comprised only the dissolved ^13^C-glucuronic acid initially, the calculated initial isotope composition of DOC in the ^13^GluC treatment is ∼8,809,000‰, and thus is not shown in panel (f). Error bars in panels (a–d,f) indicate the standard deviation calculated from triplicate incubation bottles, while for measurements of δ^13^C of the solid-phases (e), solid-phase samples from triplicate incubation bottles were homogenized and subsampled for quadruplicate analyses.

Consistent with the consumption of protons during the reductive dissolution of Fe(III) minerals under anoxic conditions,^53^ increases in pH and concomitant decreases in Eh were recorded in all treatments during the 25 °C, 6 week incubation (Figure 1a,b) and were accompanied by increases in solid-associated Fe(II) (Table S6 and Figure S7). For the control treatment, in which no ^57^Fe-ferrihydrite or ^13^C-glucuronic acid was added, the relatively high Eh recorded after 6 weeks of anoxic incubation (Eh_7_ = ∼50 mV) is similar to conditions previously recorded during anoxic incubation of iron-rich organic soils from Iceland and is attributed to the high amounts of easily reducible Fe in the soils^34^ having poised the redox potential at the Fe^3+^/Fe^2+^ redox couple and thus promoted Fe(III) as an electron acceptor. Confirming this, the addition of ^57^Fe-ferrihydrite in the ^57^Fh treatment, which increased the soil Fe content by 16.1% (Table S3), resulted in lesser changes in soil slurry pH and similar trends in Eh, suggesting that easily reducible Fe was not limiting in the soil. In contrast, the addition of ^13^C-glucuronic acid, both as dissolved (^13^GluC treatment) and coprecipitated with ^57^Fe-ferrihydrite (^57^Fh^13^GluC treatment), resulted in greater changes in both pH and Eh compared to the control or the ^57^Fh treatments. The most drastic changes were seen with the addition of dissolved ^13^C-glucuronic acid in the ^13^GluC treatment, which reached the highest pH (∼6.7), while similarly low Eh were recorded in both the ^57^Fh^13^GluC and ^13^GluC treatments (Eh_7_ ∼ -110 mV) after 6 weeks of anoxic incubation.

Trends in concentrations of aqueous Fe (Fe_aq_) and dissolved organic carbon (DOC) (Figure 1c,d) and amounts of solid-associated Fe(II) (Table S6 and Figure S7) indicate similar effects resulting from the addition of ^57^Fe-ferrihydrite and/or ^13^C-glucuronic acid. Specifically, in agreement with similar Eh values measured during the incubation, trends in Fe_aq_ and solid-associated Fe(II) were most similar for the control and ^57^Fh treatments. Yet, overall DOC release was lower in the ^57^Fh treatment, suggesting that the added ^57^Fe-ferrihydrite served as additional sorption sites, promoting the re-adsorption of released DOC. Considering that the C/Fe molar ratio of the ^57^Fe-ferrihydrite-^13^C-glucuronic acid coprecipitate added in the ^57^Fh^13^GluC treatment was 0.4; a ratio at which free mineral surface sorption sites are expected to be abundant,^45,46^ a lower release of DOC may also be expected from the ^57^Fh^13^GluC treatment. The fact that both Fe_aq_ and DOC were higher in the ^57^Fh^13^GluC treatment than in the control treatment suggests that the addition of the coprecipitated ^57^Fe-ferrihydrite did not facilitate increased DOC sorption. Instead, the coprecipitated ^57^Fe-ferrihydrite may have been more susceptible to microbial reduction due to both the increased structural distortion expected with coprecipitated minerals,^46,54^ and its close association with an easily accessible electron donor^55^ (the ^13^C-glucuronic acid). Indeed, although Fe_aq_ represents a relatively small fraction of total Fe in the system (<2%), changes in the iron isotope composition of the aqueous phase in the ^57^Fh^13^GluC treatment reveal that higher fractions of the coprecipitated ^57^Fe-ferrihydrite were found in solution after 6 weeks of anoxic incubation (Figure S6 and Table S5; *p* < 0.05). Moreover, assuming that all ^13^C-glucuronic acid added in the ^57^Fh^13^GluC treatment was initially found coprecipitated with ^57^Fe-ferrihydrite in the solid phase, consistent increases in the fraction of ^57^Fe atoms found in solution combined with decreases in δ^13^C_solid-phase_ (Figure 1e) and increases in δ^13^C_DOC_ (Figure 1f) indicate that the release of the coprecipitated ^13^C-glucuronic acid to solution was coupled to the reductive dissolution of the coprecipitated ^57^Fe-ferrihydrite. That the δ^13^C_DOC_ values remain relatively consistent in this treatment, and Fe_aq_ is enriched in ^57^Fe atoms (compared to the total system) supports the hypothesis that the formation of dissolved Fe(II/III)-organic complexes^35,56^ or a carbon-rich fine colloid fraction^34^ is responsible for retaining concomitantly released Fe in solution in this treatment. It should be noted, however, that initial desorption tests indicated that a fraction of solid-phase C in the ^57^Fe-ferrihydrite-^13^C-glucuronic acid coprecipitate was found to be rapidly soluble in UPW and therefore may contribute, in part, to the initial increase in δ^13^C_DOC_.

Overall, concentrations of Fe_aq_ and DOC were highest in the ^13^GluC treatment. Despite the initial addition of the dissolved ^13^C-glucuronic acid to the aqueous phase, DOC concentrations measured after 3 d were similar amongst all treatments, indicating the rapid sorption of the dissolved ^13^C-glucuronic acid into the soil matrix. This is further evidenced by the increased δ^13^C_solid-phase_ values (Figure 1e). Yet, sorption of the ^13^C-glucuronic acid to the soil matrix alone would not alter the isotope composition of the remaining DOC. Thus, measured changes in δ^13^C_DOC_, which decrease in the ^13^GluC treatment over time (Figure 1f), confirm the mobilization of native SOM as DOC. Combined with the high concentrations of Fe_aq_ and higher amounts of solid-associated Fe(II) recorded in this treatment compared to the control, the results suggest that the addition of dissolved ^13^C-glucuronic acid stimulated microbial activity and resulted in increased reductive dissolution of native Fe(III) minerals and the concurrent release of native MAOM as DOC. Similar indications of increased microbial reduction of Fe(III) compared to the control treatment were also seen in the ^57^Fh^13^GluC treatment. Indeed, at the first measured timepoint (3 d), solid-associated Fe(II) was higher in the ^57^Fh^13^GluC treatment compared to both the control and the ^13^GluC treatment (Table S6 and Figure S7). Yet, aside from the first 3 d of anoxic incubation, concentrations of Fe_aq_, DOC (Figure 1c,d), and/or solid-associated Fe(II) (Table S6 and Figure S7) were lower in the ^57^Fh^13^GluC treatment compared to the ^13^GluC treatment, suggesting that the stimulation of microbial Fe(III) reduction in the ^57^Fh^13^GluC treatment was limited due to the lower bioavailability of the coprecipitated ^13^C-glucuronic acid.

### Iron Addition Limits Stimulated Mineralization of Native SOM

**Figure 2:**
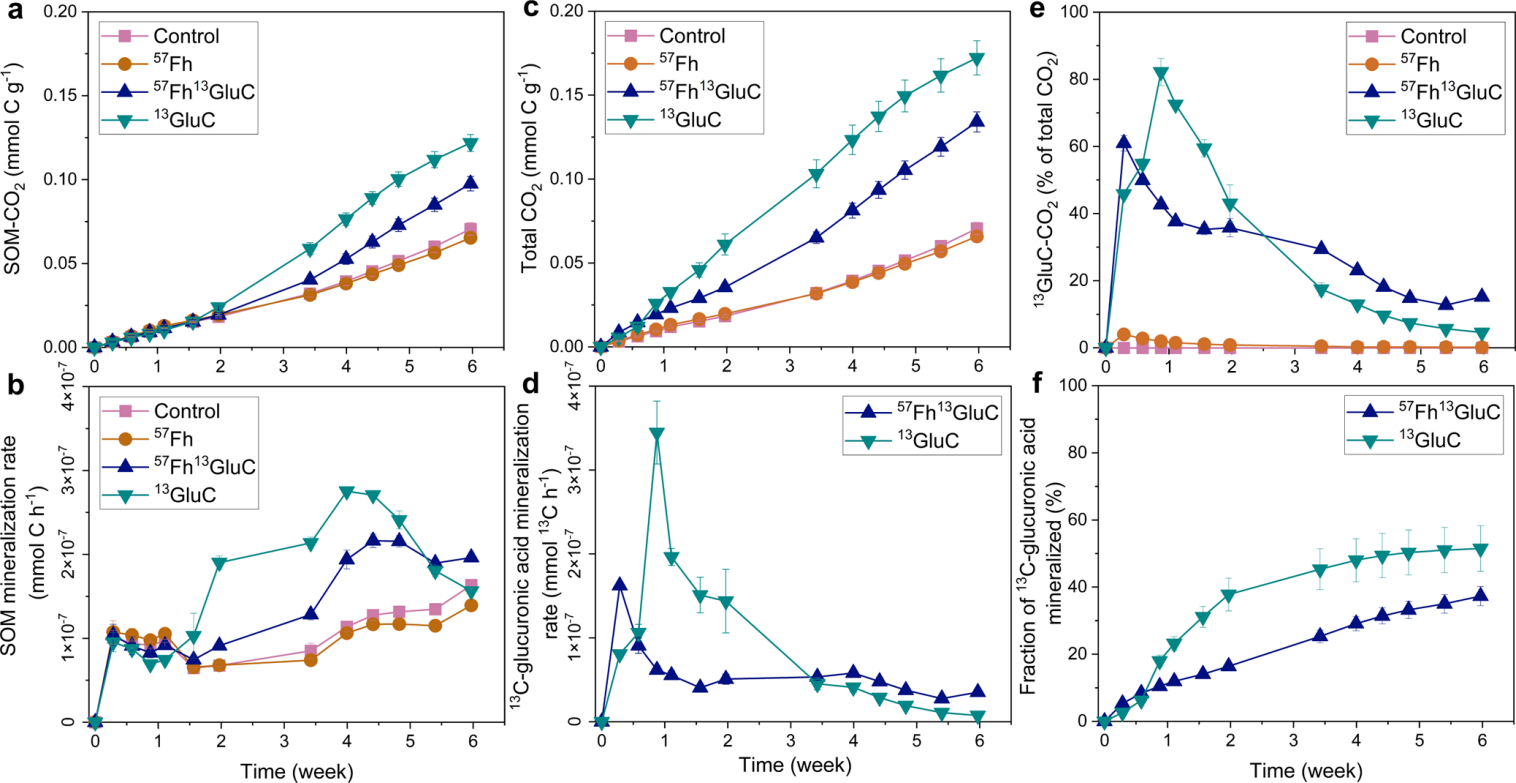
Trends in CO_2_ production in the 25 °C, 6 week anoxic incubations. Native SOM mineralized [SOM-CO_2_; panel (a)]. Mineralization rates of native SOM (b). Total CO_2_ produced, which includes SOM-CO_2_ and ^13^GluC-CO_2_; panel (c). Mineralization rates for ^13^C-glucuronic acid in the ^13^GluC and ^57^Fh^13^GluC treatments (d). Fraction of CO_2_ that derives from ^13^C-glucuronic acid (e). Estimated extent of mineralization of the added substrate (f). Error bars show the standard deviation between triplicate incubation bottles.

Accumulated CO_2_ resulting from the mineralization of native SOM (SOM-CO_2_) in the anoxic soil slurries is shown in Figure 2a. Mirroring general trends in the aqueous geochemical data (Figure 1), total amounts of SOM-CO_2_ produced in the control and ^57^Fh treatments were most similar. However, toward the end of the experiment (>4 weeks), mineralization rates of the native SOM (Figure 2b) were lower in the ^57^Fh treatment compared to the control treatment (e.g., 1.63 × 10^–7^ and 1.39 × 10^–7^ mmol C h^–1^ at 6 weeks for the control and ^57^Fh treatments, respectively, *p* < 0.05), suggesting that the additional ^57^Fe-ferrihydrite stabilized native SOM and hindered its biodegradation to CO_2_. The addition of iron minerals has been shown to inhibit soil respiration in aerobic soil incubations, whereby the mineral surfaces are thought to sorb otherwise easily decomposable substrates.^12,17^ However, the impact of iron mineral additions on CO_2_ production in anoxic soils is less clear, with increases, decreases, and no effect on CO_2_ production reported in various mineral-enriched soils.^39,40,57^ This may be explained by the complex role that Fe(III) plays in sub- or anoxic soils, where it may (1) act as a terminal electron acceptor and thereby increase CO_2_ emissions through the metabolic coupling of oxidation of OC to iron reduction^22,38^ or (2) may facilitate the sorption and stabilization of DOC, thus limiting CO_2_ emissions. In the iron-rich organic soil horizon used in this study, ferrihydrite already comprised a significant fraction of the total iron present.^34^ Recently, we showed that ferrihydrite in Icelandic wetland soils is rapidly reduced and re-precipitated during redox cycles.^34^ Thus, it is likely that the native ferrihydrite of the soil horizon included here formed in situ and therefore precipitated in the presence of abundant OM (Table S1). As such, it is likely that the native ferrihydrite showed higher structural disorder than the synthetic ^57^Fe-ferrihydrite.^46,54^ Thus, the added ^57^Fe-ferrihydrite, being more ordered than native ferrihydrite, may have sorbed and stabilized the otherwise labile organic substrates rather than acted as a terminal electron acceptor, leading to an overall inhibition of CO_2_ production. This interpretation is further supported by the aqueous geochemical data, which showed the lowest release of DOC in the ^57^Fh treatment (Figure 1).

Because the overall production of SOM-CO_2_ was hardly affected by the addition of ^57^Fe-ferrihydrite in the ^57^Fh treatment compared to the control, we ascribe the variations in iron mineral reduction and native SOM mineralization in the ^57^Fh^13^GluC and ^13^GluC treatments to the bioavailability of the added ^13^C-glucuronic acid. Mineralization rates of the native SOM increased in the ^57^Fh^13^GluC and ^13^GluC treatments after ∼1.5 weeks, resulting in a 40 and 75% increase in total SOM-CO_2_ produced in the ^57^Fh^13^GluC and ^13^GluC treatments (respectively) compared to the control treatment after 6 weeks (*p* < 0.01). The alteration of microbial decomposition of native SOM as a response to input from fresh carbon sources, termed the “priming effect”,^59^ is well documented, in particular for aerobic soil systems,^12,17,60−62^ and is influenced by both the chemical structure of the substrate^62^ as well as the amount of substrate added.^61^ Under anoxic conditions, additions of glucose have been similarly shown to increase native SOM mineralization compared to glucose-free controls.^63^ For the ^57^Fh^13^GluC treatment, the priming effect resulted in less SOM-CO_2_ being produced than in the ^13^GluC treatment (∼35%, *p* < 0.05). Limited priming effects following the addition of mineral-sorbed organic substrates (compared to mineral-free organic substrate additions) have been previously demonstrated during aerobic soil incubations and are attributed to the strong chemical interactions between the Fe mineral surface and the substrates inhibiting the bioavailability of the latter.^12,17^ Our results suggest that a similar mechanism of mineral protection of organic substrates occurs under anoxic conditions, whereby, in agreement with aqueous geochemical data, the mineral-associated ^13^C-glucuronic acid is less bioavailable, and therefore, the associated priming effect is limited. It should be noted, that in the ^57^Fh^13^GluC treatment, the ^13^C-glucuronic acid was coprecipitated with the ^57^Fe-ferrihydrite. Thus, the limited priming effect seen in our study is likely a result of both the occlusion of the ^13^C-glucuronic acid within the mineral-aggregate structure in addition to the above-mentioned chemical interactions between the mineral surface and the substrate. However, coprecipitation, rather than sorption, is thought to drive the formation of MAOM at redox interfaces.^52^ Therefore, our ^57^Fe-ferrihydrite-^13^C-glucuronic acid coprecipitate is likely an appropriate representative of naturally occurring MAOM.

### Coprecipitation with Ferrihydrite Inhibits Mineralization of Glucuronic Acid

Total CO_2_ (i.e., the sum of CO_2_ derived from the ^13^C-glucuronic acid (^13^GluC-CO_2_) and SOM-CO_2_) produced in each of the treatments is shown in Figure 2c. Generally, trends mirror those seen in SOM-CO_2_ (compare to Figure 2a). For the control and ^57^Fh treatments, the difference between total CO_2_ and SOM-CO_2_ was negligible. However, total CO_2_ in the ^57^Fh^13^GluC and ^13^GluC treatments is 22 and 40% higher than SOM-CO_2_ (respectively, *p* < 0.01), indicating that mineralization of the added ^13^C-glucuronic acid contributed significantly to overall CO_2_ production. Indeed, ^13^GluC-CO_2_ comprised the majority of total CO_2_ produced throughout the experiment in these treatments (Figure 2e), with the contribution fraction of ^13^GluC-CO_2_ to total CO_2_ peaking at ∼61 and ∼82% (measured after 48 h and 1 week of anoxic incubation in the ^57^Fh^13^GluC and ^13^GluC treatments, respectively).

The rapid onset of ^13^GluC-CO_2_ formation indicates that ^13^C-glucuronic acid was immediately available to soil microorganisms. Yet, as demonstrated by the maximum mineralization rate recorded only 1 week after substrate addition (Figure 2d), mineralization of the dissolved ^13^C-glucuronic acid in the ^13^GluC treatment was delayed. In addition to direct utilization, easily assimilable substrates are also used by microorganisms in the biosynthesis of extracellular enzymes, extracellular polysaccharides, and cell wall polymers,^64^ collectively termed here “microbial biomass”. A lag phase in substrate mineralization following substrate addition may suggest that its incorporation into microbial biomass preceded mineralization.^48^ Whereby with low substrate additions, substrate mineralization follows zero-order kinetics,^65^ with higher substrate additions, the microbial utilization capacity of the existing microbial biomass may become saturated.^61^ When applied to the results of this study, this may suggest that the rate of ^13^C-glucuronic acid addition (5410 μg ^13^C-glucuronic acid C g^–1^ soil, Table S3) saturated the microbial utilization capacity and may have initially stimulated the growth of and been incorporated into microbial biomass (contributing to increases in δ^13^C_solid-phase_; Figure 1e), followed by a slower phase of mineralization attributed to soil microbial community turnover.^48^ For the ^57^Fh^13^GluC treatment, which did not show a lag phase in substrate mineralization, applying similar reasoning suggests that a fraction of the coprecipitated ^13^C-glucuronic acid may have been easily accessible to microorganisms but was significantly small such that the utilization capacity of the existing microbial biomass was not saturated, thus leading to rapid substrate utilization and high initial ^13^C-glucuronic acid mineralization rates. The immediate bioavailability of ^13^C-glucuronic acid in the ^57^Fh^13^GluC treatment is consistent with high initial concentrations of water-soluble C derived from the ^57^Fe-ferrihydrite-^13^C-glucuronic acid coprecipitate.

Microbial utilization of the rapidly desorbed ^13^C-glucuronic acid in the ^57^Fh^13^GluC treatment likely explains the higher amounts of solid-associated Fe(II) measured in this treatment at 3 d (Table S6 and Figure S7), indicating that more microbial reduction of Fe(III) occurred in this treatment initially. Still, aqueous geochemical data indicates that conditions (Eh, pH, Fe_aq_, and DOC) in the ^57^Fh^13^GluC and ^13^GluC treatments within the first 3 d were otherwise identical and that trends in pH, Fe_aq_, and solid-associated Fe(II) continued similarly through the first week of anoxic incubation (Figure 1, Table S6, and Figure S7). Moreover, mineralization rates of the native SOM were similar among all treatments during this time (Figure 2b). Thus, attribution to a utilization-based lag phase rather than variations in aqueous geochemical conditions seems more likely to explain the delayed mineralization of the ^13^C-glucuronic acid in the ^13^GluC treatment.

In aerobic soil incubations, substrate mineralization is influenced by variations in soil mineralogy, specifically clay content and mineral surface area,^19,20^ as well as differing microbial community structures.^62^ In our study, the extent of substrate mineralization was influenced by substrate bioavailability being altered through coprecipitation with ferrihydrite. The estimated fraction of the added ^13^C-glucuronic acid that mineralized during the 6 week anoxic incubation is presented in Figure 2f. While the first 48 h likely reflect the rapid mineralization of the desorbed ^13^C-glucuronic acid from the ^57^Fe-ferrihydrite-^13^C-glucuronic acid coprecipitate, at all timepoints after 1 week, the fraction of mineralized glucuronic acid was higher in the ^13^GluC treatment (*p* < 0.05). After 2 weeks, 56% less of the added ^13^C-glucuronic acid was mineralized in the ^57^Fh^13^GluC treatment compared to the ^13^GluC treatment (*p* < 0.05). That lower amounts of ^13^C-glucuronic acid were mineralized in the ^57^Fh^13^GluC treatment at 2 weeks despite similar amounts of solid-associated Fe(II) found here (11.1 vs 10.1 mg g^–1^) strongly suggests that the limited mineralization of ^13^C-glucuronic acid is due to its coprecipitation with ^57^Fe-ferrihydrite. After 6 weeks of anoxic incubation, the difference in mineralization extent due to coprecipitation with ^57^Fe-ferrihydrite was reduced to 27% (*p* < 0.05). This is attributed to a decrease in mineralization rates of the ^13^C-glucuronic acid in the ^13^GluC treatment after ∼2 weeks, while mineralization rates of the coprecipitated ^13^C-glucuronic acid in the ^57^Fh^13^GluC treatment continued steadily for the duration of the experiment. That the fractions of solid-associated Fe(II) and ^57^Fe atoms found in Fe_aq_ similarly increased in the ^57^Fh^13^GluC treatment during this time confirms that the release and subsequent mineralization of the coprecipitated ^13^C-glucuronic acid were coupled to the reductive dissolution of the coprecipitated ^57^Fe-ferrihydrite. For the ^13^GluC treatment, the decrease in mineralization rates may indicate a shift in mineralization regimes from microbial utilization of the added substrate to slow turnover of the ^13^C incorporated into microbial biomass, or it may alternatively suggest that the ^13^C-glucuronic acid, initially added in the dissolved phase, sorbed to existing minerals in the soil matrix (Figure S2) and thus was similarly protected from biodegradation through strong chemical interactions with the mineral surfaces. Moreover, the fact that the amount of solid-associated Fe(II) continually increased in the ^13^GluC treatment without maintenance of ^13^C mineralization rates suggests that, if sorbed to the soil matrix, the ^13^C-glucuronic acid may have preferentially sorbed to aluminosilicates rather than SRO iron minerals, both of which were abundant in this soil.^34^

### Iron Protection of OC Is Enhanced at Lower Temperatures

In the 12 °C incubations, total amounts of CO_2_ derived from both the native SOM and the ^13^C-glucuronic acid were lower than in the 25 °C incubations (Figure S8c), as is expected due to decreases in microbial activity at lower temperatures.^66^ However, in contrast to the strong priming effect and increases in SOM-derived CO_2_ resulting from the ^13^C-glucuronic acid addition at 25 °C, mineralization of native SOM proceeded similarly in all treatments at 12 °C (Figure S6). As the priming effect only appeared after ∼1.5 weeks of anoxic incubation at 25 °C, a delayed onset of soil priming of >8 weeks at 12 °C is plausible. Since, at higher latitudes, soils are frozen for much of the year-Iceland, for example, has a growing season of just 3–5 months,^44^ if delayed, the impact of an eventual priming effect in similar Fe-rich, organic high latitude soils may be limited.

Trends in mineralization of the ^13^C-glucuronic acid in the ^57^Fh^13^GluC and ^13^GluC treatments proceeded similarly at 12 °C as seen at 25 °C. This includes the delay in mineralization of the dissolved ^13^C-glucuronic acid in the ^13^GluC treatment, possibly suggesting saturation of the microbial utilization capacity and the incorporation of ^13^C-glucuronic acid into microbial biomass, and higher mineralization rates in the first week of anoxic incubation in the ^57^Fh^13^GluC treatment (Figure S8d), relating to mineralization of a rapidly desorbed fraction of ^13^C-glucuronic acid from the ^57^Fe-ferrihydrite-^13^C-glucuronic acid coprecipitate. Overall, the extent of mineralization of the ^13^C-glucuronic acid was lower in both treatments at 12 °C (37% versus 51% for the ^13^GluC treatment and 16% versus 37% for the ^57^Fh^13^GluC treatment after 6 weeks in the 12 and 25 °C incubations, respectively). However, the effect of mineral protection of ^13^C-glucuronic acid through its coprecipitation with ^57^Fe-ferrihydrite was stronger, with 69–58% less of the added ^13^C-glucuronic acid mineralized in the ^57^Fh^13^GluC treatment compared to the ^13^GluC treatment over the 8 week incubation (compared to 27% less after 6 weeks of anoxic incubation at 25 °C). Assuming that the lower temperature limited microbial s resulted in less Fe(III) reduction,^66^ the larger impact of mineral protection at low temperatures seems to confirm that, in the 25 °C incubations, the continual slow release and subsequent mineralization of the coprecipitated ^13^C-glucuronic acid were coupled to the ongoing reduction of the coprecipitated ^57^Fe-ferrihydrite.

### Environmental Implications

In the absence of O_2_, microbial reduction of Fe(III) is thought to drive the release of MAOM to soil solutions^25−30^ and directly result in CO_2_ production through the metabolic coupling of oxidation of OC to Fe(III) reduction.^22,38^ However, our study demonstrates that, under anoxic conditions, mineralization of MAOM is significantly inhibited; 27 and 58% less mineral-associated ^13^C-glucuronic acid was mineralized after 6 weeks of anoxic incubation at 25 °C and after 8 weeks anoxic incubation at 12 °C, respectively. Furthermore, the iron mineral protection of the OC influenced the mineralization of the native SOM in that the priming effect resulting from the addition of ^13^C-glucuronic acid was decreased by 35% in the 6 week, 25 °C incubations. Thus, this work constitutes quantifiable evidence that iron mineral protection of organic carbon occurs under reducing soil conditions via mechanisms widely recognized in oxic soils, namely, OC sorption to and occlusion within mineral aggregate structures comprising SRO iron minerals.^4,6,8−12^ The existence of active iron mineral protection mechanisms under reducing soil conditions may help explain the abundant storage of Fe-bound OC in soil and sediments, which are exposed to periodic reducing conditions. However, ongoing Fe reduction during the incubation period led to the continued slow release and mineralization of the MAOM. This is particularly clear for the 25 °C incubations, where trends in the fraction of the coprecipitated ^13^C-glucuronic acid mineralized did not reach a plateau within the experiment timeframe (6 weeks, Figure 2f). This suggests that the iron mineral protection mechanisms studied here are likely not sufficient to explain the existence and age of Fe-bound OC in temperate soils and sediments that are permanently reduced. In the 12 °C, 8 week incubations, a clear plateau was also not reached (Figure S8f), yet mineralization rates declined after 6 weeks, suggesting that, at lower temperatures, MAOM may persist for longer.

That the extent of iron mineral protection as well as the priming effect associated with the substrate additions varied with incubation temperature offers insight into both current and potential future trends in carbon cycling in soils. In general, mineralization rates of both the ^13^C-glucuronic acid and the native SOM were lower at low temperatures (12 °C) than at high temperatures (25 °C), where increased microbial reduction of Fe(III) simultaneously led to loss in effectiveness of iron mineral protection over time. This suggests that Fe-bound C stored in high latitude anoxic soils and sediments, where temperatures remain significantly cooler for longer periods of time, may be comparatively more stable than MAOM stored in temperate or tropical soils and sediments. Moreover, the demonstration of a delayed priming effect following substrate addition at 25 °C and no measurable priming effect recorded within 8 weeks at 12 °C suggests that native SOM in high latitude soils may be less vulnerable following the addition of fresh labile substrates. Collectively, the temperature-based effectiveness of iron mineral protection indicates that increases in soil temperature due to, e.g., climate change are likely to impact both the mineralization of MAOM as well as native SOC storage and mobility, although from this study, which included only two temperatures, we cannot conclude whether the warming of high latitude or temperate/tropical soils and sediments may be more greatly affected.

Finally, compared to pure mineral phases, Fe(III)–OC coprecipitates vary in structure and may be more vulnerable to reductive dissolution.^54,55,67^ A recent study by Chen et al.^52^ demonstrated that SRO iron minerals produced through the abiotic oxidation of aqueous Fe(II) in soil solution in the presence of DOC were more susceptible to reductive dissolution, serving as electron acceptors and promoting CO_2_ production upon subsequent exposure to anoxic conditions. Higher reductive dissolution of the coprecipitated ^57^Fe-ferrihydrite compared to the pure ^57^Fe-ferrihydrite was also recorded in our study; however, the addition of the pure ^57^Fe-ferrihydrite did not result in increased CO_2_ production. This finding contrasts with the results of similar anoxic incubation studies, which report increased CO_2_ production in soils following the addition of pure ferrihydrite or goethite, where it is suggested that Fe(III) in these minerals acted as electron acceptors.^39,40^ However, in the soil used here, easily reducible native iron was abundant (7.3 wt % Fe_T_, Table S1, ∼31.7 mg g^–1^ Fe_O_^34^). Thus, it is plausible that, owing to the high native ferrihydrite content, microbial reduction of both the coprecipitated and pure ^57^Fe-ferrihydrite was less than if an iron-poor soil was used. This suggests that the magnitude of iron mineral protection may also depend on native soil characteristics, including (iron) mineral content, composition, and crystallinity.

## Supporting Information Available

Soil profile description, mineral synthesis and characterization, and aqueous Fe isotope data (PDF)

## Notes

The datasets generated and/or analyzed during the current study are available from the corresponding author on reasonable request. Other data are included in the Supporting Information.
