# Supplementary material for: Coprecipitation with Ferrihydrite Inhibits Mineralization of Glucuronic Acid in an Anoxic Soil
Source: Environ Sci Technol. 2023 Jun 9;57(25):9204–13. doi: 10.1021/acs.est.3c01336 (PMC10308808; doi:10.1021/acs.est.3c01336)
Supplement: Supplementary file 1 — es3c01336_si_001.pdf [file es3c01336_si_001.pdf]

Supporting Information to:

'Coprecipitation with ferrihydrite inhibits mineralization of glucuronic acid in an anoxic soil'

Laurel K. ThomasArrigo,*[a] Sophie Vontobel,[a] Luiza Notini,[a] Tabea Nydegger,[a]

[a]*Soil Chemistry Group, Institute of Biogeochemistry and Pollutant Dynamics, Department of Environmental Systems Science, ETH Zurich, Universitätstrasse 16, CHN, CH-8092 Zurich, Switzerland*

(15 Pages, 8 Figures, 6 Tables)

Table of contents

1. Soil profile location, description, and mineralogy S2
2. Mineralogic characterization of the isotope-labelled (co)precipitates S4
3. Experimental conditions S7
4. Details to aqueous DOC isotope ratio measurements S8
5. Details to headspace gas measurements S9
6. Iron isotope composition of the aqueous Fe(II) S10
7. Acid-extractable Fe(II) S12
8. Native SOM mineralization in the low temperature experiments S13
9. References S14

*Corresponding author: laurel.thomas@unine.ch

1. Soil profile location, description, and mineralogy

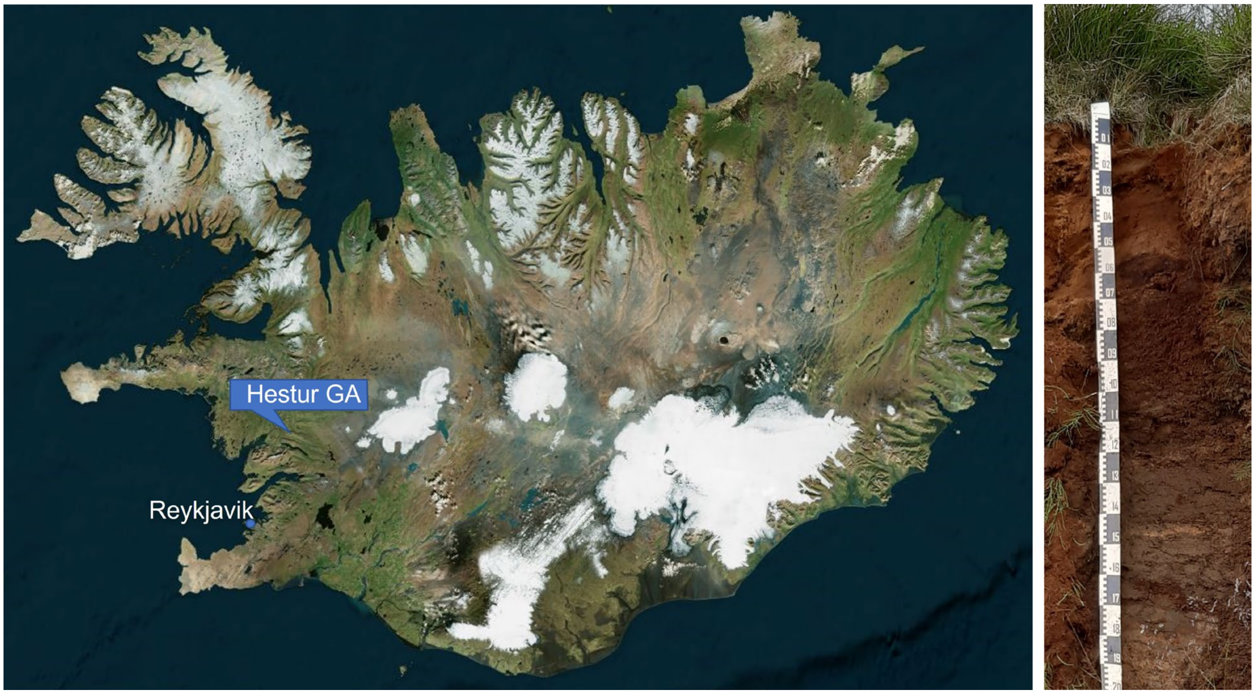

Figure S1. (L) Map of Iceland with soil profile sampling location. Orthoimage based on data from National Land Survey of Iceland. (R) The complete Hestur_GA (2020) soil profile.

In the Borgarfjörður catchment in western Iceland, basalts are primarily Tertiary (older than 3.1 Ma)[1] and the region receives a low influx of aeolian deposition of volcanic ash (25-100 g m^{-2} yr^{-1}).[2] The mean annual temperature (MAT) is 4.6 °C with average annual precipitation of 988 mm yr^{-1} (station Hvanneyri, 2002–2020; Iceland Meteorological Office, IMO). The Hestur_GA site represents an example of the drainage-impacted low lying (<200 m elevation) wetlands typical across north and western Iceland.[3]

Soils for characterization and the incubation study were collected in July 2020. The soil profile was described following FAO guidelines.[4] Individual horizons were manually homogenized and packaged in their field-moist state into plastic bags which were then stored at 4°C in the dark. Subsets of each soil horizon were air dried (30°C) and sieved (<2 mm, nylon) for characterization. Soil pH was determined after re-suspending the dried soil in UPW at a solid:solution ratio of 1:5 for 1 hr. Total element contents of each soil horizon were measured with energy-dispersive X-ray fluorescence (XRF) spectrometry (Spectro X-Lab 2000) and total C and N contents with an elemental analyzer (Vario MAX Cube, Elementar).

Mineral composition of the soil horizon was determined by powder X-ray diffraction (XRD, D8 Advance, Bruker). For these analysis, 30 °C dried and sieved soil was milled to ~50 µm using a disk swing mill. Milled soil material was analyzed as powder XRD in Bragg−Brentano

geometry using Cu K$\alpha_{1,2}$ radiation (λ = 1.5418 Å, 40 kV, and 40 mA) and a high-resolution energy-dispersive 1-D detector (LYNXEYE). Diffractograms were recorded from 10° to 70°2θ with a step size of 0.02°2θ and 6 s acquisition time per step. The relative contributions of the crystalline mineral phases in the diffraction patterns were determined by Rietveld Quantitative Phase Analysis (QPA) using the TOPAS software (Version 5, Bruker AXS) in combination with published crystallographic structure files. Additionally, the amount of amorphous material was estimated by the internal standard method in the TOPAS software using aluminum oxide (Al_2O_3, Fluka) as the internal standard mixed into the soil at a mass ratio of 1:2 (Al_2O_3:soil).

Compared to the same soil profile sampled in 2019, (Hestur_GA in ref. 5), the depth of soil horizons in the 2020 soil profile varied slightly, reflecting the heterogeneity of soils affected by cryoturbation. Still, physical, elemental, and mineralogic characteristics of the soil horizon selected for this incubation study, positioned at 60-72 cm depth, were most similar to the soil horizon Hestur_GA_45-60 used in ref. 5 (compare to values reported in Table S1). Soil pH was 4.56 and total Fe and C contents were 73.1 mg g^{-1} and 21.6 wt.%, respectively. X-ray diffraction patterns indicated the presence of plagioclase feldspars, pyroxenes, and small contributions from quartz and contained a significant amorphous fraction (Figure S2 and Table S2).

Table S1. Physical and elemental characterization of the Hestur_GA (2020) soil profile.

Site Name	Depth[a] (cm)	Horizon	pH (H_20)[b]	C_T[c] (wt.%)	C/N[c] Mass ratio	T[d] Al (mg g^{-1})	T[d] Fe (mg g^{-1})	T[d] Si (mg g^{-1})
Hestur_GA (2020)	0-50	A		11.7	11.8	32.2	193.1	77.7
	50-60	O1		29.2	15.7	26.8	62.8	49.7
	**60-72**	**O2**	**4.56**	**21.6**	**15.9**	**30.7**	**73.1**	**66.0**
	72-80	B1		9.7	13.9	79.3	75.2	131.3
	80-100	O3		25.4	16.8	44.9	54.6	78.6
	100-120	O4		11.6	14.7	77.8	77.7	115.4
	120-180	B2		3.2	10.9	94.1	96.2	164.4
	180-200	B3		6.3	16.7	49.8	216.8	106.0
	>200	O5		32.5	16.6	24.8	69.3	38.0

[a]Below soil surface. [b]Measured in suspended soils (1:5 solid:solution ratio) after 1 h at room temperature. [c]Determined with an elemental analyzer (CNS). [d]Total element content (XRF). The horizon used in this study is shown in bold.

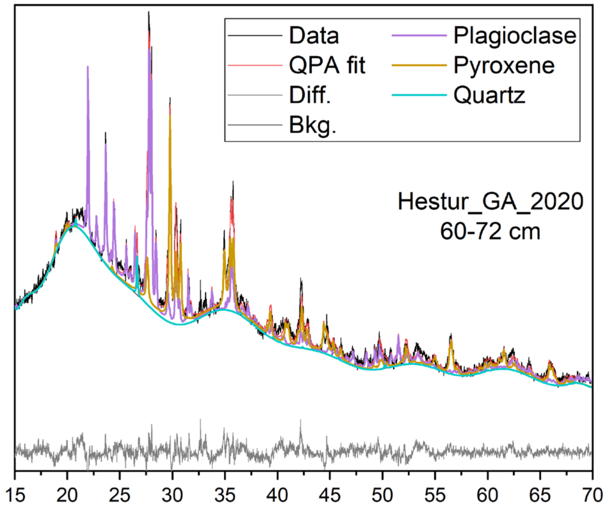

Figure S2. X-ray diffraction pattern and Rietveld Quantitative Phase Analysis of the soil horizon used in this study. Fit parameters are detailed in Table S2. Black lines show the measured data, red lines the QPA fit, the dark gray line directly under the pattern shows the background, and the lower light gray line indicates the model misfit. Additional colored lines (purple = plagioclase, gold = augite, teal = quartz) represent the fitted contribution from each respective mineral phase.

Table S2. Mineral contributions determined by Rietveld QPA of XRD pattern of soil horizon.

Site Name	Depth (cm)	R_{wp}[a]	GOF[b]	Plagioclase[c]	Pyroxene[d]	Quartz	Amorphous material[e]
				(%)			
**Hestur_GA (2020)**	60-72	4.03	1.88	53	46	2	58

[a]Weighted profile R-factor. [b]Goodness of Fit is defined as R_{wp}/R_{exp}, where R_{wp} is the weighted profile R-factor and R_{exp} the expected R-factor. [c]Comprised of anorthite sodian with small (<10%) contributions from albite, andesine, anorthoclase and labradorite. [d]Comprised of augite with small (<2%) contributions from diopside. [e]Determined using the internal standard method with Al_2O_3 as the internal standard. Published structure files: anorthite sodian (ICSD 9287),[6] albite (COD 2107372),[7] andesine (ICSD 66126),[8] anorthoclase low (ICSD 31180),[9] labradorite (ICSD 100232),[10] augite (ICSD 16905),[11] diopside (AMCSD 0005234),[12] quartz (AMCSD 0000789).[13]

2. Mineral synthesis and characterizations

All solutions used in this experiment were prepared from ultra pure water (UPW, Milli-Q®, Millipore, 18.2 MΩ·cm). Synthesis of isotope-labelled ferrihydrite (^{57}Fh) and the ferrihydrite-glucuronic acid coprecipitate (^{57}Fh^{13}GluC) followed previously published methods[14-16] with modifications included to enable synthesis of ^{57}Fe-labelled minerals from Fe(0) metal powder. To this end, an ^{57}Fe(III) stock solution was prepared by dissolving ^{57}Fe(0) metal powder (95.08% ^{57}Fe, Isoflex, USA) in 2 M HCl (Normatron®, VWR) followed by oxidation with H_2O_2. For the synthesis of ^{57}Fh, the ^{57}Fe(III) stock solution was titrated with 1 M NaOH (Titrisol®) under vigorous stirring (1200/min) until a pH of 7.1 ± 0.1 was reached. For the ferrihydrite coprecipitate, we chose to work with glucuronic acid; low molecular weight organic acid with a single carboxyl

group (Figure S3). Being a derivative of glucose, a high energy substrate that can be rapidly utilized by soil microorganisms,[17] mineralization of glucuronic acid is expected to be similarly rapid. For the synthesis of ^{57}Fh^{13}GluC, ^{13}C-labelled glucuronic acid (^{13}GluC, 99% x(^{13}C), D-[UL-$^{13}C_6$]glucuronic acid sodium salt monohydrate, Omicron Biochemicals) was equilibrated overnight in darkness in UPW water adjusted to pH 7.0 with 1 M NaOH under vigorous stirring (1200/min). The ^{13}C-glucuronic acid-containing solution was then acidified to pH 4.0 with 1 M HNO_3 (Normatron®, VWR) and purged with N_2(g) for 15 min. After adding an aliquot of the ^{57}Fe(III) stock solution, the solution was titrated to pH 7.1 ± 0.1 with 1 M NaOH as described in the synthesis of ferrihydrite. The mineral suspensions were then centrifuged at 3600 *g* for 15 minutes, decanted, and re-suspended in UPW three times until the conductivity of the supernatant was <350 µS/cm. Afterwards, the suspensions were shock frozen by dropwise injection into liquid N_2 and freeze dried, homogenized with a mortar and pestle, and stored in brown glass bottles in a desiccator until use.

Total Fe content of the (co)precipitates was determined after acid dissolution and subsequent analysis with inductively coupled plasma-optical emission spectrometry (ICP-OES, Agilent 5100). Total C content of ^{57}Fh^{13}GluC was measured with an elemental analyzer (CHNS-932, LECO; n = 4). The Fe:C molar ratio of the ferrihydrite-glucuronic acid coprecipitate ^{57}Fh^{13}GluC was 0.42. The fraction of easily-desorbed C in the coprecipitate was determined after re-suspending ~4 mg ^{57}Fh^{13}GluC in 1 mL of UPW and setting on an orbital shaker (150 rmp) for 4 hours followed by centrifugation (18620 rcf for 10 min) and measuring of the supernatant for dissolved organic carbon (DOC) as described below. Results showed that ~10 mg g^{-1} C was extracted by H_2O, accounting for ~22% of total C in the coprecipitate. The mineral composition of the (co)precipitates was confirmed by powder XRD. For these analyses, dried sample material (~10 mg) was resuspended in ethanol (~30 µL, Merck) and pipetted onto a polished silicon wafer (Sil'tronix Silicon Technologies, France). Diffractograms were recorded as described above however using a step size of 0.02°2θ and 10 s acquisition time per step. For both ^{57}Fh and ^{57}Fh^{13}GluC, XRD patterns confirmed the presence of 2-line ferrihydrite, visible as broad maxima around 2.54 and 1.49 Å (Figure S4).

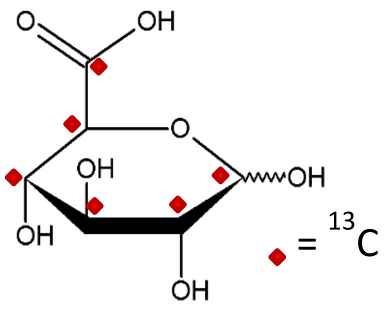

Figure S3. Chemical structure of glucuronic acid used in this study. Mw: 194 Da.

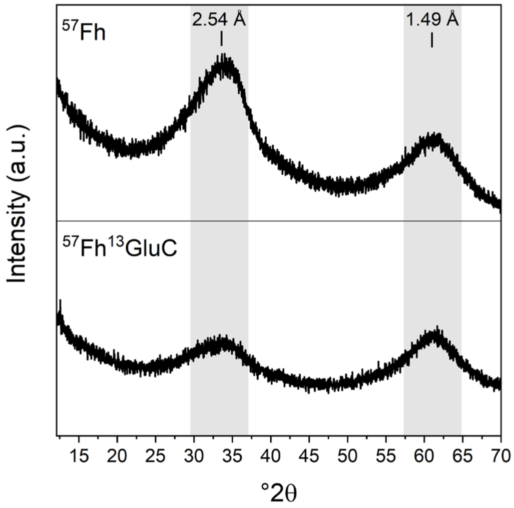

Figure S4. X-ray diffraction patterns of ^{57}Fh and ^{57}Fh^{13}GluC. (Co)precipitates show the broad peak features at 2.54 and 1.49 Å typical of 2-line ferrihydrite.

3. Experimental conditions

Table S3. 25°C incubation experimental conditions.[a]

Treatment Name	Dry[b] soil weight	Total[c] native Fe	Total[d] native ^{57}Fe	Total[e] native C	Total[f] native ^{13}C	Spike[g]	^{57}Fe added	Increase in total soil Fe content	Total system[h] Fe isotope fractions ^{56}Fe / ^{57}Fe	Mössbauer signal from added ^{57}Fe	^{13}C added	Increase in total soil C content	Change in total system δ^{13}C
	(g per bottle)	(mg per bottle)						(%)	(%)	(%)	(mg)	(%)	(‰)
**Control**	3.5	256	5.42	756	8.09	0	0	0	97.8 / 2.3	0	0	0	0
	1.5	109	2.31	324	3.46	0	0	0	97.8 / 2.3	0	0	0	0
**^{57}Fh**	3.5	256	5.42	756	8.09	81.08	45	16.1	83.1 / 16.9	89.3	0	0	0
	1.5	109	2.31	324	3.46	34.77	19.3	16.1	83.1 / 16.9	89.3	0	0	0
**^{57}Fh^{13}GluC**	3.5	256	5.42	756	8.09	88.06	45	16.1	83.1 / 16.9	89.3	4.09	0.45	+506.1
	1.5	109	2.31	324	3.46	37.77	19.3	16.1	83.1 / 16.9	89.3	1.76	0.45	+506.1
**^{13}GluC**	3.5	256	5.42	756	8.09	11.37	0	0	97.8 / 2.3	0	4.09	0.45	+506.1
	1.5	109	2.31	324	3.46	4.88	0	0	97.8 / 2.3	0	1.76	0.45	+506.1

[a]Soil:water ratio 1:10. Experiments were conducted in triplicate. [b]Two complete sets of treatments were prepared to allow for (1) destructive sampling of the soil slurry (3.5 g dry weight, 117 mL experiment bottles) and (2) sampling of headspace gasses only (1.5 g dry weight, 58 mL experiment bottles). [c]Based on XRF. [d]Based on natural Fe isotope abundance (f^{57}Fe = 2.12%).[18] [e]Based on an elemental analyzer (CNS). [f]Based on natural C isotope abundance (f^{13}C = 1.07%).[19] [g]Either ^{57}Fh, ^{57}Fh^{13}GluC, or ^{13}GluC. [h]Considering ^{56}Fe and ^{57}Fe iron isotopes only.

Table S4. 12°C incubation experimental conditions.[a]

Treatment Name	Dry[b] soil weight	Total[c] native Fe	Total[e] native C	Total[f] native ^{13}C	Spike[g]	^{57}Fe added	Increase in total soil Fe content	^{13}C added	Increase in total soil C content	Change in total system $\delta^{13}C$
	(g per bottle)	(mg per bottle)					(%)	(mg)	(%)	(‰)
**Control**	3.0	219	648	6.93	0	0	0	0	0	0
**^{57}Fh**	3.0	219	648	6.93	51.53	28.6	13.0	0	0	0
**^{57}Fh^{13}GluC**	3.0	219	648	6.93	55.97	28.6	13.0	2.60	0.41	+375.6
**^{13}GluC**	3.0	219	648	6.93	8.70	0	0	2.60	0.41	+375.6

[a]Soil:water ratio 1:10. Experiments were conducted in duplicate. [b]Sample bottles were used to collect and measure headspace gases only. [c]Based on XRF. [d]Based on natural Fe isotope abundance ($f^{57}Fe$ = 2.12%).[18] [e]Based on an elemental analyzer (CNS). [f]Based on natural C isotope abundance ($f^{13}C$ = 1.07%).[19] [g]Either ^{57}Fh, ^{57}Fh^{13}GluC, or ^{13}GluC.

4. Details to aqueous DOC and solid-phase C isotope ratio measurements

The isotopic ratio of aqueous samples expressed as $\delta^{13}C_{DOC}$ was measured by converting the DOC into CO_2 by oxidation with a heated $Na_2S_2O_8$ solution followed by measurement of its isotope ratios. For data normalization and quality control, two in-house reference materials (C_3 and C_4 sugars) and potassium hydrogen phthalate (KHP) were measured in each run. The external reproducibility of control standards was better than 0.2‰ ($\pm 1\sigma$).

At selected time-points, the $\delta^{13}C$ value of solid-phase samples was measured using a ThermoFisher Flash-EA 1112 coupled with a Conflo IV interface to a ThermoFisher Delta V isotope ratio mass spectrometer (IRMS). The individual sample batches were normalized to the V-PDB scale using the internal laboratory standards Atropin ($\delta^{13}C$ = -21.10 ‰) and ‘‘Glucose-enriched” ($\delta^{13}C$ = + 62.09 ‰) which were calibrated with NBS22 ($\delta^{13}C$ = -30.03) and IAEA CH-6 ($\delta^{13}C$ = -10.46). Reproducibility of the measurements is better than 0.2‰. Samples were measured in quadruplicate. Isotope ratios are reported in the conventional δ-notation with respect to the Vienna Pee Dee Belemnite (V-PDB) standard:

$$\delta^{13}C = ((^{13}C/^{12}C)_{sample}) / (^{13}C/^{12}C)_{VPDB} - 1) * 1000$$

5. Details to headspace gas measurements

For the 25°C incubation headspace gas sampling, ~25 mL of headspace gas was removed through a needle connected to a syringe with a 3-way stopcock valve and immediately injected into the SSIM. While measurements were running (detailed below), the headspace of the bottles was purged with humidified N_2 gas at a flow rate of 750 mL min^{-1} for 10 minutes. During the purging, the bottles were placed on an orbital shaker (150 rpm) at room temperature. Bottles were then injected with ~30 mL of humidified N_2 gas through a needle connected to a syringe with a 3-way stopcock valve prior to being returned to an orbital shaker (150 rpm) in a temperature-controlled room (25 °C). For the 12°C incubation headspace gas sampling, an aliquot of headspace gas was removed through a needle connected to a syringe with a 3-way stopcock valve and injected into a 10 mL N_2-flushed sealed septum vial for CO_2 and CH_4 concentration measurements with gas chromatography. An additional aliquot of headspace gas was injected into a 30 mL N_2-flushed sealed septum vial for $\delta^{13}C$ analysis with CRDS.

Headspace gas samples injected into the SSIM achieved a pressure of >1100 Torr (1170 ± 23, $\bar{x} \pm \sigma$) and measurements were conducted using N_2 as a carrier gas. Samples were measured over an 8-minute measurement cycle. For all treatments, temperatures, and timepoints, production of CH_4 was negligible. Concentrations of CO_2 from the ^{57}Fh and Control treatments were quantified relative to a 5-point calibration curve of CO_2 standards of known concentrations (400, 500, 700, 2600, 5000 ppm, Messer and SPECIALTY) and the $\delta^{13}C$ values were corrected (value and drift) based on two certified CO_2 tank standards (-3‰ and -36‰, Carbagas). Concentrations of CO_2 and the ^{13}C atom fraction of CO_2 from the ^{57}Fh^{13}GluC and ^{13}GluC treatments were calibrated using 11 standards varying from 1 to 85% $x(^{13}C)$. These standards were created by mixing 98% $x(^{13}C)$ Na_2CO_3 (Sigma Aldrich) with natural abundance Na_2CO_3 ($\delta^{13}C$ = 1.42‰, Fluka), digesting with an excess of 12M HCl and removing aliquots of headspace.[20] These standards were used to confirm the accuracy and precision of measuring CO_2 concentrations at high delta values using CRDS. To this end, each of the 11 standards (1 to 85 % $x(^{13}C)$) were prepared as three different concentrations ranging from ~400 ppm to ~6000 ppm CO_2 and were measured both with CRDS and GC as described above. In addition, the total CO_2 concentrations in selected experiment samples were additionally measured with GC. The linearity of the $\delta^{13}C$ correction at varying CO_2 concentrations and high $\delta^{13}C$ values (R^2 = 0.9997) as well as the linear relationship between total CO_2 concentrations at varying $\delta^{13}C$ values measured with CRDS and GC (R^2 = 0.9985) are shown Figure S5 and confirm the viability of using CRDS for both CO_2 concentration as well as $\delta^{13}C$ value determination in the ranges of this study.

Dissolved CO_2 in the soil slurries was estimated based on Henry's law using the dimensionless H constant. Therefore, in the Control and ^{57}Fh treatments, where pH remained <6 for the duration of the incubation, estimates of dissolved CO_2 are likely accurate, whereas increases in pH >6 and thus a dominance of bicarbonate in solution in the ^{57}Fh^{13}GluC and ^{13}GluC treatments (after 5 and 4 weeks, respectively) indicate that dissolved CO_2 may be underestimated at these timepoints.

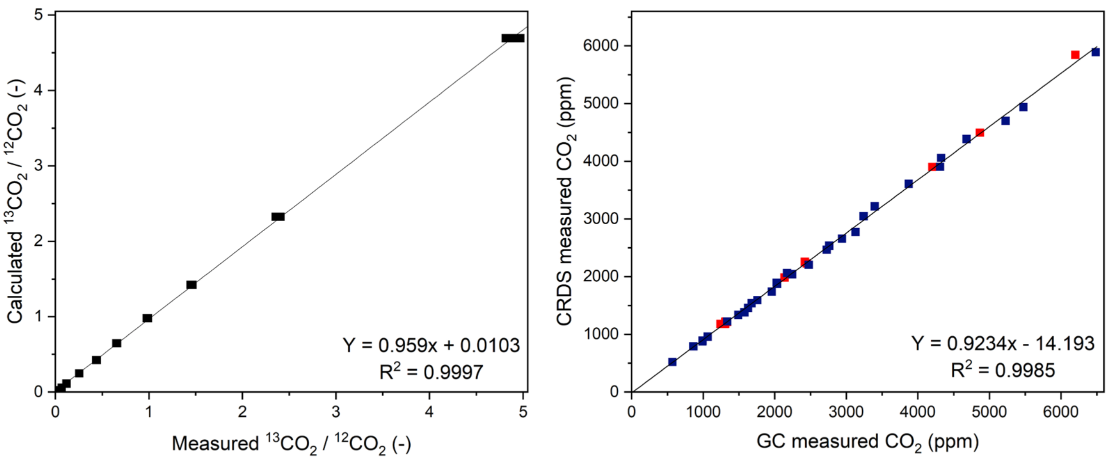

Figure S5. Calculated versus measured $^{13}CO_2$ / $^{12}CO_2$ molar ratios (left panel, n = 37) and total CO_2 concentrations measured by CRDS and GC (right panel, n= 42). In both panels, measured samples include synthetic standards (n = 11, 1 to 85% $x(^{13}C)$; each standard prepared at 3 different CO_2 concentrations ranging from ~400 ppm to ~6000 ppm, randomly measured in duplicate). Additionally, for selected experiment samples (n = 12), total CO_2 concentrations were measured with both CRDS and GC (shown in red in the right panel).

6. Iron isotope composition of the Fe_{aq}

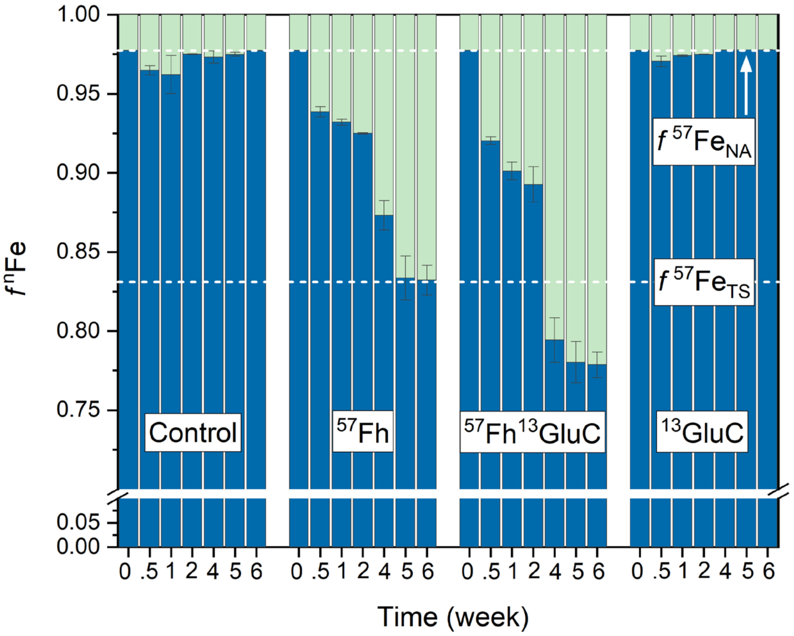

Figure S6. Iron isotope composition of Fe_{aq}, shown as $f\,^{n}Fe$, where n = 56 (blue) or 57 (green), for the 25°C, 6-week incubations. Dashed lines are: the calculated isotope composition of the total system (TS) following the addition of the isotope-labelled coprecipitates and the natural abundance (NA) isotope composition considering the iron isotopes ^{56}Fe and ^{57}Fe only.[18] Error bars show the standard deviation from triplicate incubation bottles.

Table S5. Absolute amounts and iron isotope composition of Fe_{aq} in the 25°C, 6-week incubations.

**Treatment**	**Time**	**^{56}Fe**	**^{57}Fe**	**Fraction of added ^{57}Fh in solution**	**Fraction of total Fe in solution**
	**(week)**	**(mg)**	**(mg)**	**(%)**	**(%)**
**^{57}Fh**	initial	0	0	0	0
	3 d	0.106	0.007	0.014	0.04
	1 week	0.102	0.007	0.015	0.04
	2 week	0.230	0.017	0.038	0.08
	4 week	0.913	0.116	0.257	0.35
	5 week	1.257	0.209	0.464	0.51
	6 week	2.329	0.391	0.869	0.94
**^{57}Fh^{13}GluC**	initial	0	0	0	0
	3 d	0.071	0.006	0.013	0.03
	1 week	0.073	0.007	0.016	0.03
	2 week	0.374	0.040	0.089	0.14
	4 week	1.403	0.289	0.641	0.60
	5 week	1.987	0.436	0.970	0.86
	6 week	3.020	0.669	1.486	1.31

7. Acid extractable Fe(II)

Table S6. Solid-associated Fe(II) in the 25°C, 6-week incubations.

**Treatment**	**Time**	**0.5 M HCl extractable Fe(II)**[a]
	**(week)**	**(mg g^{-1})**
**Control**	3 d	2.5
	1 week	2.9 (0.8)
	2 week	3.6 (1.5)
	4 week	7.7 (0.8)
	5 week	10.9 (1.4)
	6 week	15.0 (0.8)
**^{57}Fh**	3 d	3.3
	1 week	4.0 (0.5)
	2 week	5.3 (0.7)
	4 week	7.7 (0.6)
	5 week	8.8 (0.5)
	6 week	12.6 (0.5)
**^{57}Fh^{13}GluC**	3 d	8.0
	1 week	6.3 (0.3)
	2 week	11.1 (0.8)
	4 week	12.7 (1.1)
	5 week	14.8 (0.3)
	6 week	18.9 (1.2)
**^{13}GluC**	3 d	3.6
	1 week	9.4 (6.7)
	2 week	10.1 (0.2)
	4 week	18.2 (1.3)
	5 week	20.1 (1.9)
	6 week	23.6 (1.4)

[a]As determined by the 1,10-phenanthroline method[21, 22] in 0.5 M HCl extracts.[23] Errors in parenthesis represent the standard deviation of triplicate incubation bottles. For 3 d samples, triplicate solid-phases were combined.

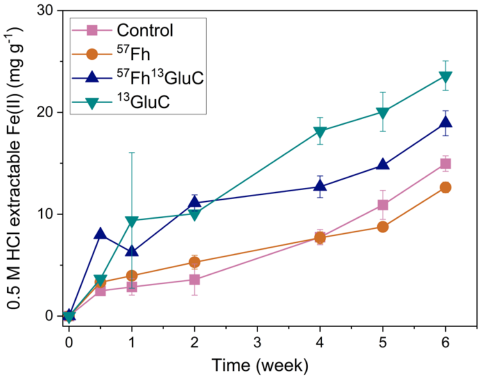

Figure S7. Trends in solid-associated Fe(II) in the 25 °C, 6-week incubation. Fe(II) concentrations were determined by the 1,10-phenanthroline method[21, 22] in 0.5 M HCl extracts.[23] Errors in parenthesis represent the standard deviation of triplicate incubation bottles. For 3 d samples, triplicate solid-phases were combined.

8. Native SOM and ^{13}C-glucuronic acid mineralization in the 12°C incubation.

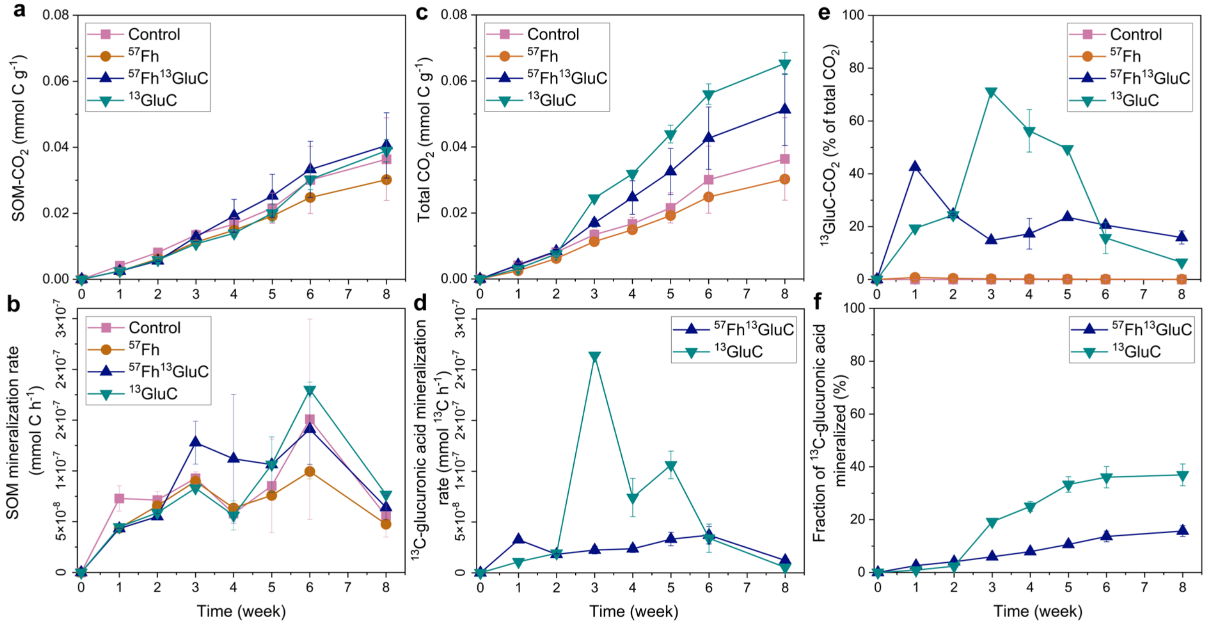

Figure S8. Trends in CO_2 production in the 12°C, 8-week anoxic incubations. (a) Native SOM mineralized (SOM-CO_2). (b) Mineralization rates of native SOM. (c) Total CO_2 produced, which includes SOM-CO_2 and ^{13}GluC-CO_2. (d) Mineralization rates for ^{13}C-glucuronic acid in the ^{13}GluC and ^{57}Fh^{13}GluC treatments. (e) Fraction of CO_2 that derives from ^{13}C-glucuronic acid. (f) Estimated extent of mineralization of the added substrate. Error bars show the range of duplicate incubation bottles.

8. References

(1) Harðarson, B. S.; Fitton, J. G.; Hjartarson, A. Tertiary volcanism in Iceland. *Jokull* **2008**, *58*, 161-178.
(2) Arnalds, O. Dust sources and deposition of aeolian materials in Iceland. *Iceland. Agr. Sci.* **2010**, *23*, 3-21.
(3) Arnalds, O. *The Soils of Iceland*; Springer Science+Business Media, 2015.
(4) WRB, I. W. G. World Reference Base for Soil Resources 2014. International soil classification system for naming soils and creating legends for soil maps. FAO, Rome. **2014**.
(5) ThomasArrigo, L. K.; Kretzschmar, R. Iron speciation changes and mobilization of colloids during redox cycling in Fe-rich, Icelandic peat soils. *Geoderma* **2022**, *428*, 116217.
(6) Toman, K.; Frueh, A. J. On the centrosymmetry of intermediate plagioclases. *Z. Kristallogr.* **1973**, *138*, 337-342.
(7) Ribbe, P. H.; Megaw, H. D.; Taylor, W. H.; Ferguson, R. B.; Traill, R. J. The albite structures. *Acta Crystall. B-Stru.* **1969**, *B 25*, 1503-1518.
(8) Fitz Gerald, J. D.; Parise, J. B.; Mackinnon, I. D. R. Average structure of an AN_{48} plagioclase from the Hogarth ranges. *Am. Miner.* **1986**, *71*, 1399-1408.
(9) Harlow, G. E. The anorthoclase structures - The effects of temperature and composition. *Am. Miner.* **1982**, *67*, 975-996.
(10) Wenk, H. R.; Joswig, W.; Tagai, T.; Korekawa, M.; Smith, B. K. The average structure of An 62-66 labradorite. *Am. Miner.* **1980**, *65*, 81-95.
(11) Peacor, D. R. Refinement of crystal structure of a pyroxene of formula M_{II} M_{III} $(Si_{1.5}Al_{0.5})O_6$[1]. *Am. Miner.* **1967**, *52*, 31-41.
(12) Raudsepp, M.; Hawthorne, F. C.; Turnock, A. C. Evaluation of the Rietveld method for the characterization of fine-grained products of mineral synthesis; The diopside-hedenbergite join. *Can. Mineral.* **1990**, *28*, 93-109.
(13) Levien, L.; Prewitt, C. T.; Weidner, D. J. Structure and elastic properties of quartz at pressure. *Am. Miner.* **1980**, *65*, 920-930.
(14) ThomasArrigo, L. K.; Byrne, J.; Kappler, A.; Kretzschmar, R. Impact of organic matter on iron(II)-catalyzed mineral transformation in ferrihydrite-OM coprecipitates *Environ. Sci. Technol.* **2018**, *52*, 12316-12326.
(15) ThomasArrigo, L. K.; Kaegi, R.; Kretzschmar, R. Ferrihydrite growth and transformation in the presence of ferrous Fe and model organic ligands. *Environ. Sci. Technol.* **2019**, *53*, 13636-13647.
(16) Schwertmann, U.; Cornell, R. M. *Iron Oxides in the Laboratory: Preparation and Characterization*; WILEY-VCH Verlag GMBH & Co. KGaA, 2000.
(17) Hill, P. W.; Farrar, J. F.; Jones, D. L. Decoupling of microbial glucose uptake and mineralization in soil. *Soil Biol. Biochem.* **2008**, *40*, 616-624.
(18) Taylor, P. D. P.; Maeck, R.; Debievre, P. Determination of the absolute isotopic composition and atomic weight of a reference sample of natural iron. *Int. J. Mass Spectrom.* **1992**, *121*, 111-125.
(19) De Laeter, J. R.; Bohlke, J. K.; De Bievre, P.; Hidaka, H.; Peiser, H. S.; Rosman, K. J. R.; Taylor, P. D. P. Atomic weights of the elements: Review 2000 - (IUPAC technical report). *Pure and Applied Chemistry* **2003**, *75* (6), 683-800.
(20) Creamer, C. A.; de Menezes, A. B.; Krull, E. S.; Sanderman, J.; Newton-Walters, R.; Farrell, M. Microbial community structure mediates response of soil C decomposition to litter addition and warming. *Soil Biol. Biochem.* **2015**, *80*, 175-188.
(21) Loeppert, R. H.; Inskeep, W. P. Iron. In *Methods of Soil Analysis, Part 3. Chemical Methods*, Sparks, D. L., Page, A. L., Helmke, P. A., Loeppert, R. H., Soltanpour, P. N., Tabatabai, M. A., Johnston, C. T., Sumner, M. E. Eds.; Soil Science Society of America, 1996; pp 639-644.
(22) Fadrus, H.; Malý, J. Supression of iron(III) interference in determination of iron(II) in water by 1,10-

phenanthroline method. *Analyst* **1975**, *100* (1193), 549-554.
(23) Tishchenko, V.; Meile, C.; Scherer, M. M.; Pasakarnis, T. S.; Thompson, A. Fe^{2+} catalyzed iron atom exchange and re-crystallization in a tropical soil. *Geochim. Cosmochim. Acta* **2015**, *148*, 191-202.
